# High Mobility Group Box 1 Influences HSV1716 Spread and Acts as an Adjuvant to Chemotherapy

**DOI:** 10.3390/v10030132

**Published:** 2018-03-15

**Authors:** Leslee Sprague, Joel M. Lee, Brian J. Hutzen, Pin-Yi Wang, Chun-Yu Chen, Joe Conner, Lynne Braidwood, Kevin A. Cassady, Timothy P. Cripe

**Affiliations:** 1 The Ohio State University College of Medicine, Biomedical Sciences Graduate Program, Columbus, OH 43210, USA; les.sprague@nationwidechildrens.org (L.S.); joel.lee@nationwidechildrens.org (J.M.L.); kevin.cassady@nationwidechildrens.org (K.A.C.); 2 Nationwide Children’s Hospital, Division of Hematology/Oncology/BMT and Center for Childhood Cancer and Blood Diseases, Columbus, OH 43205, USA; brian.hutzen@nationwidechildrens.org (B.J.H.); Pinyi.wang@nationwidechildrens.org (P.-Y.W.); Chun-yu.chen@nationwidechildrens.org (C.-Y.C.); 3 Virttu Biologics, BioCity Glasgow, Newhouse ML1 5UH, UK; Joe.conner@virttu.com (J.C.); Lynne.braidwood@virttu.com (L.B.); 4 Nationwide Children’s Hospital, Division of Infectious Diseases and Center for Childhood Cancer and Blood Diseases, Columbus, OH 43205, USA

**Keywords:** oncolytic virus, virus induced therapeutic adjuvant, conditioned media, herpes simplex virus

## Abstract

High Mobility Group Box 1 (HMGB1) is a multifunctional protein that plays various roles in the processes of inflammation, cancer, and other diseases. Many reports document abundant HMGB1 release following infection with oncolytic viruses (OVs). Further, other groups including previous reports from our laboratory highlight the synergistic effects of OVs with chemotherapy drugs. Here, we show that virus-free supernatants have varying cytotoxic potential, and HMGB1 is actively secreted by two established fibroblast cell lines (NIH 3T3 and 3T6-Swiss albino) following HSV1716 infection in vitro. Further, pharmacologic inhibition or genetic knock-down of HMGB1 reveals a role for HMGB1 in viral restriction, the ability to modulate bystander cell proliferation, and drug sensitivity in 3T6 cells. These data further support the multifactorial role of HMGB1, and suggest it could be a target for modulating the efficacy of oncolytic virus therapies alone or in combination with other frontline cancer treatments.

## 1. Introduction

Oncolytic viruses (OVs) are a burgeoning class of anti-cancer therapies derived from a variety of viruses and attenuated to be safe to the host yet still target and kill tumor cells [1]. The therapeutic potential that OVs demonstrate in a variety of preclinical models is being realized with the FDA approval of the first-in-class OV, T-Vec (Imylgic, Amgen) [2,3]. HSV1716 is another herpes simplex virus (HSV)—based OV with a long track record of use in preclinical and clinical models of cancer and is attenuated through the deletion of both copies of the RL1 gene which encodes for the neurovirulence factor ICP34.5 [4,5,6,7,8,9]. OVs target cancers through directly infecting and ultimately killing cancer cells, as well as activating innate and adaptive immune responses targeting both tumor and virus [10,11]. Viral replication and ultimately host-cell lysis leads to the abundant release of immunostimulatory cytokines and damage associated molecular patterns (DAMPs) that potentiate an immune response against tumor and virus alike. One of the most abundantly secreted DAMPs following oncolytic virus infection is high mobility group box 1 (HMGB1) [12,13,14,15,16,17,18].

HMGB1 is involved with many cellular processes, and its function depends on its location [19,20]. For example, nuclear HMGB1 is known to associate with chromatin and function in DNA repair processes [21,22,23,24]. Activation of the interferon pathway can trigger the translocation of HMGB1 from the nucleus to the cytoplasm [25]. In the cytosol, HMGB1 acts as a DNA sensor and associates with toll-like receptors (TLRs) [26,27]. In the extracellular space, HMGB1 can bind to and bolster the downstream effects of signaling through cytokine and nucleic acid receptors [28,29]. HMGB1 acts as a potent immunostimulatory molecule and chemoattractant for monocytic cellular infiltration during respiratory syncytial virus infection [30]. Inflammatory cell death processes such as necroptosis are characterized by active secretion of HMGB1 from dying cells, which has been previously reported with herpes simplex virus-based OVs [31].

Strategies combining OVs with chemotherapeutic drugs yield encouraging results in preclinical models, and are the subject of multiple ongoing clinical trials [32,33,34]. We have previously reported that HSV1716 combined with the small molecule Aurora A Kinase inhibitor MLN8237 acts synergistically both in vitro and in vivo using models of neuroblastoma and malignant peripheral nerve sheath tumors (MPNSTs) [7]. We postulated the synergy of HSV1716 with MLN8237, or other cytotoxic therapies, is mediated through an unknown death factor we termed “virus-induced therapeutic adjuvant” (VITA) [7,35]. We had previously found that multiple HSV1716-infected cancer cell lines produced conditioned media that augments the cytotoxicity of chemotherapy drugs on treatment naïve cancer cell lines [7,35]. However, this HSV1716 conditioned media was rarely cytotoxic and did not produce any effects on bystander cells in the absence of chemotherapy drugs. Interestingly, we found that HSV1716-infected 3T6 Swiss albino fibroblasts produced a potent bystander effect, and the conditioned media alone was cytotoxic to treatment naïve cancer cell lines [35]. Based on previous reports highlighting abundant secretion of HMGB1 following OV infection, and the many roles HMGB1 can play in cellular sensing of viruses, cytokines, and nucleic acids, we theorized that HMGB1 might contribute to bystander effects produced by 3T6 cells. To test this hypothesis, we produced HSV1716gfp conditioned media using 3T6 cells with and without pharmacologic or genetic modification of HMGB1. Here we show that virus-free conditioned media from HSV1716gfp-infected 3T6 Swiss albino cells is cytotoxic to naïve fibroblast and tumor cell lines. We show HSV1716 spreads more rapidly in 3T6 Swiss albino cells in the absence of HMGB1. We also show that HMGB1 depleted HSV1716-conditioned media is less toxic to naïve tumor cells compared to HSV1716gfp-conditioned media with abundant HMGB1. Finally, we show that HMGB1 knockdown decreases cell sensitivity to HSV1716gfp and/or doxorubicin treatment.

## 2. Materials and Methods

### 2.1. Cell Culture

NIH-3T3, 3T6-Swiss albino, S462TY, 67C-4, HEK-293T, SK-N-AS, and Vero cells were maintained in Dulbecco’s Modified Eagle’s Medium (DMEM) supplemented with 10% fetal bovine serum (FBS) and 1% penicillin/streptomycin (Gibco, Life Technologies, Eugene, OR, USA). The identity of all human cell lines used was confirmed by STR genotyping. Cells were monitored periodically for mycoplasma contamination (SouthernBiotech, Birmingham, AL, USA) and fresh cell aliquots were used after every 15 passages. 67C-4 murine MPNST cells were a kind gift from Dr. Nancy Ratner.

### 2.2. Drugs, Inhibitors Proteins, and Virus

Doxorubicin and Glycyrrhizin were resuspended in DMSO (all Sigma-Aldrich, St. Louis, MO, USA). Recombinant HMGB1 (Sigma-Aldrich, St. Louis, MO, USA) was resuspended in PBS. HSV1716gfp was used for virus studies as described previously [36]. In brief, HSV1716gfp is derived from HSV strain 17+ with deletions of both copies of the RL1 gene encoding for the neurovirulence factor ICP34.5 (HSV1716). Green fluorescent protein (GFP) is added to the RL1 gene locus and driven by the phosphoglycerate kinase (PGK) promoter [37].

### 2.3. Cell Secretion Studies

Fresh cell supernatants were analyzed for the presence of HMGB1 (IBL International, Hamburg, Germany), murine IFN-⍺ (PBL Assay Science, Piscataway, NJ, USA), murine IFN-β (R&D Systems, Minneapolis, MN, USA), murine IL-1β (Peprotech, Rocky Hill, NJ, USA), and oncostatin M (R&D systems, Minneapolis, MN, USA) by ELISA according to individual manufacturer specifications. Adenosine triphosphate (ATP) concentration was determined using the ATP Determination Kit (Invitrogen, Life Technologies, Eugene, OR, USA) according to manufacturer’s directions. All supernatants were assayed immediately following treatment avoiding freeze/thaw.

### 2.4. Virus Quantification

HSV1716 titration was performed using a standard plaque formation assay as described previously [38]. Vero cells were allowed to reach 80–90% confluence and incubated (37 °C 5% CO_2_) with serial dilutions of virus or infected cell lysates for a total of 1.5 h with rocking every 15 min. After 1.5 h of incubation, overlay media (MEM, 1% carboxymethyl cellulose, 10% FBS, 1% penicillin/streptomycin) was added to each well and incubated for 3 days at 37 °C 5% CO_2_. On day 3, the wells were aspirated and stained with crystal violet prior to enumeration.

### 2.5. Cell Proliferation, Viral Spread, and Toxocity Studies

Cell proliferation, virus spread, and cytotoxicity studies were carried out using the IncuCyte Zoom live cell microscopy and analysis system (Essen Bioscience, Ann Arbor, MI, USA). Briefly, cells were seeded on flat 96-well polystyrene tissue culture plates (Corning Costar, Corning, NY, USA) at a sub-confluent density for proliferation studies (2000–5000 cells per well) or allowed to reach 80–90% confluence before adding virus for viral spread studies. Multiple images (10× magnification) per well were captured every 3–4 h until cells reached 100% confluence or predetermined endpoint. For cytotoxicity studies, IncuCyte Cytotox Red Reagent was added to wells and the number of dead cells enumerated using the IncuCyte Zoom software (Essen Bioscience, Ann Arbor, MI, USA).

### 2.6. Conditioned Media

Conditioned media was prepared as described previously [7]. Briefly, cells were allowed to reach 80–90% confluence, and counted to ensure proper multiplicity of infection (MOI). Cells were infected with or without HSV1716 or UV-inactivated HSV1716 and incubated at 37 °C 5% CO_2_ for 24 h. The conditioned media was harvested under sterile conditions and centrifuged at 4000× *g* for 10 min. Then, supernatants were filtered once using a 0.22 µm Steriflip filter (EMD Millipore, Darmstadt, Germany) followed by a final filtration using a 0.1 µm filter (Sartorius AG, Goettingen, Germany). Conditioned media from virus infected cell lines underwent plaque forming assay to ensure complete virus removal.

### 2.7. Western Blot

For protein analysis, cells were lysed using Pierce RIPA Buffer supplemented with Protease Inhibitor Cocktail (Thermo Fisher Scientific, Rockford, IL, USA). Protein concentration was determined using the Micro BCA Protein Assay Kit (Thermo Fisher Scientific, Rockford, IL, USA) and equal protein was loaded to pre-cast NuPage Bis-Tris 4–12% protein gels (Life Technologies, Carlsbad, CA, USA). Protein was transferred to nitrocellulose membranes and probed with antibodies against HMGB1 (ab18256, Abcam, Cambridge, MA, USA), Cleaved Poly ADP- Ribose Polymerase (PARP) (Asp214, Cell Signaling Technology, Danvers, MA, USA), and β-Actin (13E5, Cell Signaling Technology, Danvers, MA, USA).

### 2.8. shRNA and Lentivirus

Depletion of HMGB1 was achieved using lentivirus-mediated shRNA. The HMGB1 gene was targeted with the shRNA sequence: CCGGATGCAGCTTATACGAAGATAACTCGAGTTATCTTCGTATAAGCTGCATTTTTTG inserted into pLKO.1 vector (Sigma-Aldrich, St. Louis, MO, USA). Lentivirus was produced by transfecting HEK-293T cells with a cocktail containing psPAX2 (Addgene plasmid #12260), pCMV-VSV-G (Addgene plasmid #8454, and pLKO.1 shHMGB1 or pLKO.1 shSCR (scramble; Addgene plasmid #17920) transfer plasmids [39,40]. HEK-293T cultures were grown in T-25 flasks (Thermo Fisher Scientific, Rockford, IL, USA) until approximately 90% confluent and transfected with the plasmid cocktail to produce supernatant containing lentivirus. Lentivirus supernatants were collected at 48 and 72 h post transfection and filtered through 0.45 µm syringe filter (Advanced Microdevices Pvt. Ltd., Ambala Cantt, India) and stored at −80 °C until use.

### 2.9. Statistical Analysis

All experiments were repeated at least three times and figures are representative of those data. Graphs were produced using GraphPad Prism (GraphPad Software Inc., La Jolla, CA, USA). Data were determined to be significant based on one-way or two-way ANOVA where applicable. Statistical differences were determined significant using the 95% confidence interval.

## 3. Results

### 3.1. Conditioned Media from 3T6 Swiss Albino Cells Is Cytotoxic to Bystander Cells

**Figure 1:**
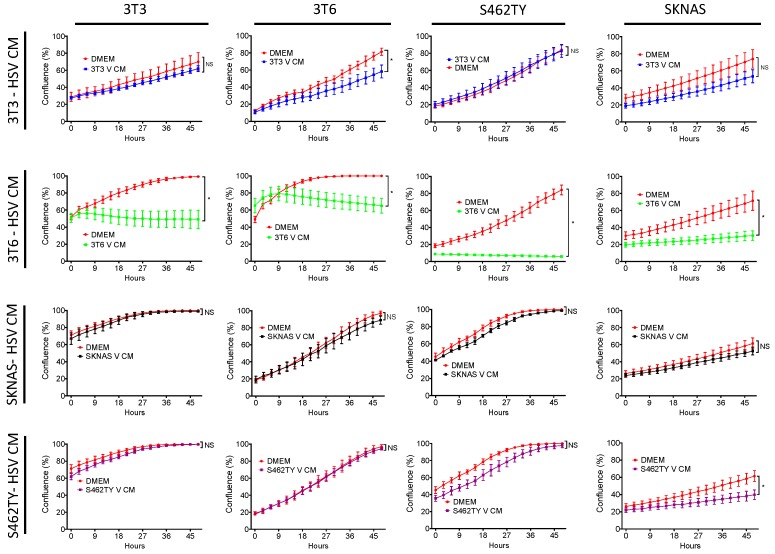
Effect of virus-free HSV1716gfp conditioned media on bystander cell proliferation. Supernatants from HSV1716gfp infected (3–5 MOI) cell lines were harvested and processed to remove virus. Resulting conditioned media (HSV CM) from each cell line (3T3, 3T6, S462TY, SK-N-AS) was added to each respective cell line. Cell proliferation was monitored over a 48-h period. Data are represented as mean ± SD. Statistical significance was determined by two-way ANOVA (Sidak’s Multiple Comparisons Test). * *p* < 0.05. Abbreviations: NS, not significant; MOI, multiplicity of infection; SD, standard deviation; DMEM, Dulbecco’s modified eagle medium; V CM, HSV1716gfp conditioned media (processed to remove virus).

We had previously found that several cell lines produced conditioned media that augmented chemotherapy toxicity following HSV1716 infection. However, we wanted to determine if conditioned media was toxic in the absence of chemotherapy drugs. To determine if HSV conditioned media was toxic to treatment naïve cells, we infected 3T3 cells, 3T6 cells, S462TY human MPNST, and SK-N-AS human neuroblastoma cells with HSV1716gfp at MOI 3 for 24 h. After 24 h, conditioned media was harvested and processed to remove any residual HSV1716gfp (V CM). Naïve 3T3, 3T6, S462TY, and SK-N-AS cells were then incubated in the presence of 100% conditioned media sourced from various cell lines or fresh DMEM (10% FBS) (Figure 1). 3T3 V CM, S462TY V CM, and SK-N-AS V CM have minimal effects on cell proliferation and are often not significantly different from cell grown in DMEM (10% FBS). However, 3T6 V CM has a significant antiproliferative effect on every cell line tested (Figure 1, second row).

### 3.2. HSV1716 Infection in NIH-3T3 and 3T6 Swiss Albino

**Figure 2:**
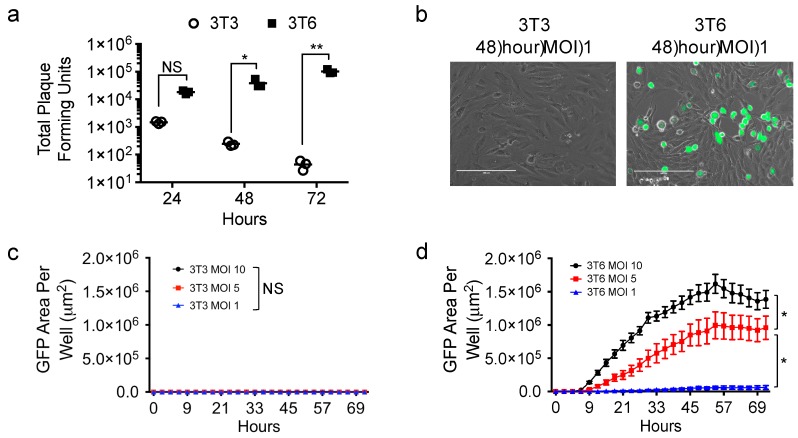
HSV1716gfp virus infection in 3T6 Swiss albino and NIH-3T3 cells. (**a**) Standard plaque assay was used to determine the amount of virus recovery from 3T3 or 3T6 cells 24, 48, and 72 h after HSV1716 infection (MOI 1); (**b**) images of 3T6 and 3T3 cells show HSV infection (GFP positive) in 3T6 while no GFP expression is observed in 3T3 cells 48 h-post infection with HSV1716gfp (MOI 1). Scale bar = 200 µm; (**c**) 3T3 or (**d**) 3T6 cells were infected at multiple MOIs with HSV1716gfp and monitored for GFP gene transfer by time lapse microscopy. Results are representative of at least 3 independent experiments. Error bars represent standard deviation. Statistical significance was determined by two-way ANOVA (Sidak’s multiple comparisons test). * *p* < 0.05, ** *p* < 0.01. Abbreviations: NS, not significant; MOI, multiplicity of infection, GFP, green fluorescent protein.

We showed cytotoxic effects of virus-free conditioned media from 3T6 Swiss albino cell lines on tumor cells that was absent in conditioned media from NIH-3T3 and two other cell lines (Figure 1), but how virus infection played a role in that disparity was unknown. Therefore, we sought to compare the infectivity of 3T6 fibroblast cells with their 3T3 cell counterpart (Figure 2). We infected 3T3 or 3T6 cells with HSV1716 at a MOI of 1 and collected cells for plaque formation assay to determine the amount of virus recovered from each cell line over 24, 48, and 72 h. 3T6 cells showed increasing virus titer over time, while 3T3 cells showed decreasing virus recovery over time (Figure 2a). Using a GFP expressing variant of HSV1716 (HSV1716gfp), we examined GFP expression by live cell fluorescent microscopy (Figure 2b). We determined that only 3T6 (Figure 2d) but not 3T3 cells (Figure 2c) showed readily quantifiable GFP expression following HSV1716gfp infection. Further, the area of GFP expression in 3T6 cells increased as the MOI increased.

### 3.3. Active HMGB1 Release Following HSV1716 Infection

**Figure 3:**
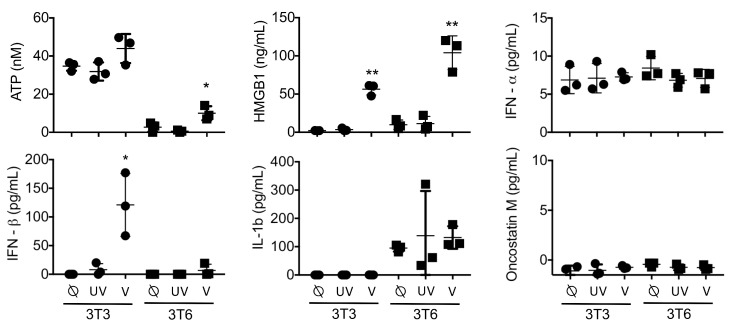
Analysis of supernatants: 3T3 or 3T6 cells with no treatment (⍉) or following infection with 3 MOI UV-Inactivated HSV1716gfp (UV) or 3 MOI HSV1716gfp (V). Levels of Interferon-β were significantly increased in 3T3 cells infected with HSV1716gfp, while both 3T3 and 3T6 cell lines secreted high levels of HMGB1 protein following infection with replication competent virus. UV inactivated HSV1716gfp did not alter cytokine profiles compared to uninfected controls. Results are representative of three independent experiments. Horizontal bars represent means. Statistical significance was determined by one-way ANOVA (Tukey’s Multiple Comparisons Test). α = 0.05; * *p* < 0.05, ** *p* < 0.01. Abbreviations: ATP, adenosine triphosphate; HMGB1, high mobility group box 1; IFN, interferon’ MOI, multiplicity of infection.

We hypothesized that differences in HSV1716 infectivity between NIH-3T3 cells and 3T6 Swiss albino would be reflected by differences in secretion of soluble factors in the conditioned media. To test this hypothesis, we screened supernatants from infected cell cultures 24 h post-HSV1716gfp infection (MOI 3) for candidate molecules including adenosine triphosphate (ATP), HMGB1, type 1 interferon (IFN-⍺, IFN-β), interleukin 1-β (IL-1β), and oncostatin M (Figure 3). Based on the supernatant screening, we found that both NIH-3T3 and 3T6 Swiss albino cells secreted ATP and HMGB1 following infection with replication-competent HSV1716gfp, but neither were detectable with UV-inactivated HSV1716gfp. 3T6 cells showed approximately two-fold higher secretion of HMGB1 compared to 3T3 cells (104 ng/mL versus 56 ng/mL, respectively). NIH-3T3 cells actively secreted IFN-β after virus infection, whereas 3T6 Swiss albino did not secrete type 1 interferon following HSV1716gfp infection. Neither NIH 3T3 or 3T6 Swiss albino secrete IL-1β or oncostatin M at baseline or after virus infection.

### 3.4. HMGB1 Inhibition Enhances Virus Spread

**Figure 4:**
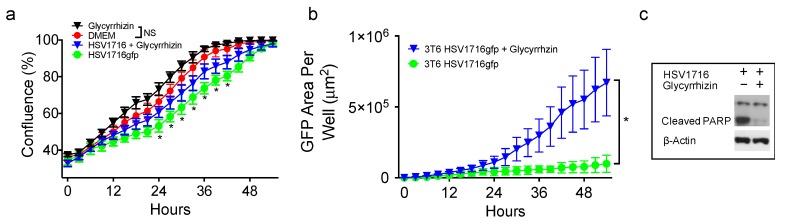
A small molecule HMGB1 inhibitor (Glycyrrhizin; 75 µM) increases HSV1716gfp spread and reduces HSV1716gfp-mediated cell death. (**a**) 3T6 cells were treated with or without glycyrrhizin (75 µM) in the presence or absence of 0.5 MOI HSV1716gfp and proliferation was monitored until reaching confluence by time lapse microscopy. Asterisks represent statistical significance between 3T6 cells treated with HSV1716gfp + glycyrrhizin versus HSV1716gfp alone; (**b**) 3T6 cell infected with 0.5 MOI HSV1716gfp were treated with glycyrrhizin (75 µM) or DMSO control and monitored for GFP expression. Areas of GFP positivity significantly increased when cells were treated with glycyrrhizin; (**c**) Western blot of PARP cleavage revealed virus-mediated cell death was reduced by glycyrrhizin 48 h following infection with 3 MOI HSV1716gfp. +, indicates *with*; −, indicates *without*. Results are representative of three independent experiments. Statistical significance was determined by two-way ANOVA (Sidak’s Multiple Comparisons Test). (* *p* < 0.05). Abbreviations: MOI, multiplicity of infection; NS, not significant; PARP, poly ADP ribose polymerase.

Because HSV1716gfp is able to infect 3T6 Swiss albino cells, we wanted to test whether or not altering the activity of HMGB1 using the small molecule HMGB1 inhibitor glycyrrhizin would impact HSV1716gfp infection. HSV1716gfp infection decreases cell proliferation, which is partially restored by HMGB1 inhibition and significant at several time points (Figure 4a). Further, glycyrrhizin did not impact the proliferation of 3T6 cells compared to 3T6 cells treated with vehicle DMSO (Figure 4a). Following infection with HSV1716gfp, we observed a significant increase of GFP expression in 3T6 cells treated with glycyrrhizin compared with HSV1716gfp-infected 3T6 cells alone (Figure 4b). We postulated that HMGB1 secretion in response to virus infection could serve as a cytotoxic means of restricting virus infection. To determine the effect of glycyrrhizin on cell death, we probed cell lysates for cleaved PARP 24 h after HSV1716gfp infection (MOI 3). We observed a marked decrease in the amount of cleaved PARP from HSV1716gfp-infected 3T6 cells in the presence of glycyrrhizin (75 µM) (Figure 4c).

### 3.5. HMGB1 Knock down Enhances Virus Spread

**Figure 5:**
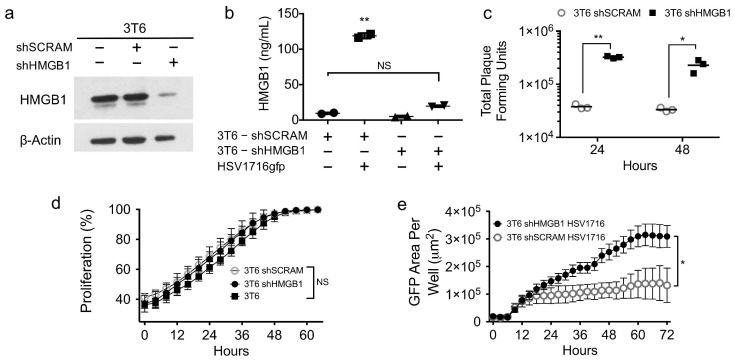
Genetic depletion of HMGB1 increases incidence and recovery of HSV1716gfp infection in 3T6 cells. (**a**) Western blot for HMGB1 in total cell lysates from parental 3T6 cells, 3T6 shSCRAM, or 3T6 shHMGB1 cells. (**b**) HMGB1 ELISA of cell supernatants from 3T6 shSCRAM or 3T6 shHMGB1 cells infected with HSV1716gfp (MOI 3) 24 h after infection. (**c**) Virus recovery from HSV1716gfp-infected 3T6 shSCRAM or shHMGB1 cells 24 and 48 h after infection (MOI 1). (**d**) Cell proliferation in HMGB1 knockdown 3T6 cells compared to parental 3T6 cell line or shSCRAM control. (**e**) Area of GFP-positive cells observed in 3T6 shHMGB1 cells infected with HSV1716gfp compared to HSV1716gfp-infected 3T6 shSCRAM cells. Data are shown as mean ± SD. +, indicates *with*; −, indicates *without*. Statistical significance was determined by two-way ANOVA (Sidak’s multiple comparisons test). α = 0.05; * *p* < 0.05, ** *p* < 0.01. Abbreviations: MOI, multiplicity of infection; SD, standard deviation.

To rule out off-target effects of glycyrrhizin, we next sought to employ a genetic approach to confirm whether HMGB1 plays a role in HSV1716gfp spread in 3T6 cells. Using shRNA targeting HMGB1, we successfully depleted HMGB1 from 3T6 cells in both total cell lysates (Figure 5a) and in cell supernatants following HSV1716gfp infection (Figure 5b). We did not observe significant differences in HGMB1 secretion in the supernatants of uninfected 3T6 shScramble control cells compared with HSV1716gfp-infected 3T6 shHMGB1 cells, underscoring the efficiency of HMGB1 depletion by shRNA. Furthermore, HMGB1 depletion did not alter cell growth kinetics compared to the 3T6 parental or 3T6 shScramble cell lines (Figure 5d). HMGB1 depletion by shRNA yielded similar results as the small molecule inhibitor glycyrrhizin with a significant increase in GFP expression in HSV1716gfp-infected HMGB1 knock-down 3T6 cells compared to shScramble controls (Figure 5e). Importantly, virus recovery correlated with the increased GFP expression with approximately one log more virus recovered from 3T6 shHMGB1 cells compared to 3T6 shScramble cells 24 and 48 h after HSV1716 infection (MOI 1) (Figure 5c).

### 3.6. HMGB1 Influences Drug Sensitivity and Bystander Cell Proliferation

**Figure 6:**
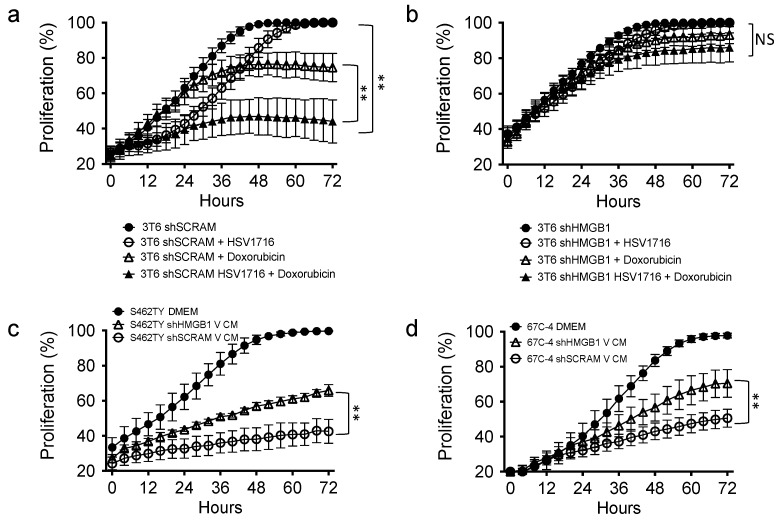
HMGB1 plays a role in the anti-proliferative capability of combination HSV1716gfp with doxorubicin treatment and bystander cell proliferation. (**a**) 3T6 cells grown in the presence of HSV1716 (MOI 2), doxorubicin (50 nM), or combination were compared to untreated counterparts; (**b**) shHMGB1 3T6 cells grown in the presence of HSV1716 (MOI 2), doxorubicin (50 nM), or combination were compared to untreated counterparts; (**c**) human MPNST (S462TY) or (**d**) murine 67C-4 cells were cultured in vitro using DMEM (10% FBS), or HSV1716 Conditioned Media (V CM) from 3T6 shScramble or shHMGB1 infected with HSV1716gfp (MOI 3). Data are representative of 3 independent experiments and represented as mean ± SD. Statistical significance was determined by two-way ANOVA (Sidak’s Multiple Comparisons Test). NS: Not Significant; ** *p* < 0.01. Abbreviations: MOI, multiplicity of infection; SD, standard deviation; CM, conditioned media; MPNST, malignant peripheral nerve sheath tumor; V CM, virus-free HSV1716gfp conditioned media.

The ability of oncolytic viruses to synergize with cytotoxic drugs has been well documented [7,17,33,41]. To determine if HMGB1 influences cell sensitivity to cytotoxic therapies, we treated 3T6 Swiss albino cells with or without HMGB1 knockdown (shHMGB1 or shSCRAM) with HSV1716gfp (MOI 2) in the presence or absence of the antineoplastic agent doxorubicin (50 nM). We observed an additive antiproliferative effect in shScramble 3T6 cell lines treated with HSV1716gfp and/or doxorubicin (Figure 6a). Strikingly, in 3T6 shHMGB1 cells, we did not observe an antiproliferative effect of HSV1716gfp, doxorubicin, or combination HSV1716gfp–doxorubicin (Figure 6b). To test whether or not the virus-free conditioned media from HSV1716gfp-infected 3T6 shScramble or HSV1716gfp-infected 3T6 shHMGB1 cell lines could impact cell proliferation of naïve tumor cells, we added processed HSV1716gfp conditioned media (V CM) to S462TY human MPNST cells (Figure 6c) and 67C-4 murine MPNST cells (Figure 6d). V CM from both shHMGB1 or shScramble cell lines inhibited cell proliferation compared to DMEM (10% FBS). However, partial recovery of proliferation was observed in shHMGB1 HSV171gfp conditioned media (shHMGB1 V CM) compared to shScramble HSV1716gfp conditioned media (shSCRAM V CM) (Figure 6c,d).

### 3.7. Exogenous HMGB1 Is Cytotoxic to Multiple Cell Lines

**Figure 7:**
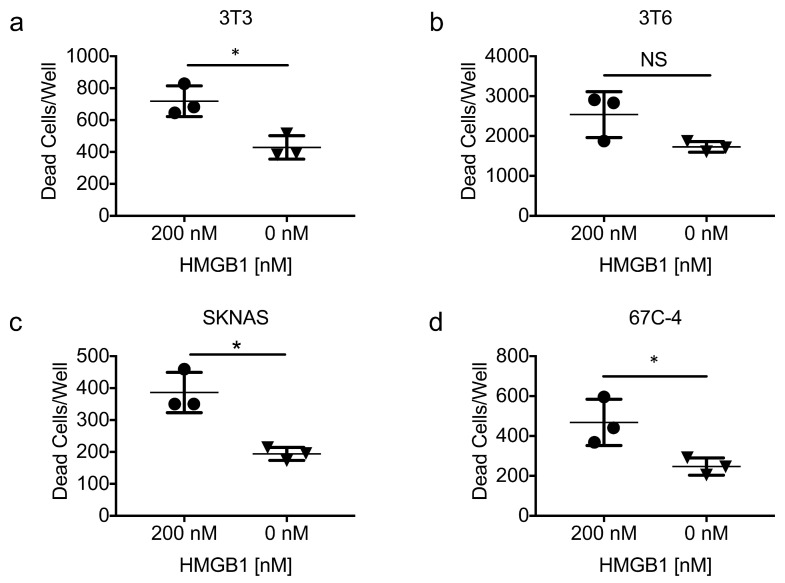
Exogenous recombinant HMGB1 is cytotoxic. (**a**) NIH-3T3 fibroblasts; (**b**) 3T6 Swiss albino fibroblasts; (**c**) SK-N-AS human neuroblastoma; and (**d**) 67C-4 murine MPNST cell lines were treated with or without recombinant human HMGB1 (rhHMGB1) protein (200 nM) for 72 h. Statistical significance was determined by unpaired two-tailed *t* test * *p* < 0.05. Error bars represent standard deviation of the mean. Abbreviations: HMGB1, high mobility group box 1; NS, not significant.

Multiple reports indicate that recombinant HMGB1 is directly cytotoxic to some cancer cell lines [42,43]. To determine if HMGB1 alone could be responsible for the antiproliferative effects of HSV1716gfp-infected 3T6 conditioned media, we added recombinant HMGB1 protein (200 nM) to multiple cell lines (3T3, 3T6, SKNAS, 67C-4) for 72 h and stained the cells with Cytotox Red (Essen Biosciences, Ann Arbor, MI, USA) to enumerate the number of dead cells in each condition. It should be noted that HMGB1 concentrations observed in HSV1716gfp-infected 3T6 cell conditioned media ranged between 85 nM and 125 nM. However, HMGB1 concentrations over 450 nM have been detected in the pleural fluid of mesothelioma patients treated with multiple doses of HSV1716 (Joe Conner; Virttu Biologics, personal communication). A significant increase in the number of dead cells was observed in the presence of recombinant HMGB1 (Figure 7a,c,d) in all but one cell line tested (3T6) (Figure 7b).

## 4. Discussion

Oncolytic viruses are an emerging class of cancer therapeutics, which are finding utility in the clinic and continue to be investigated in clinical trials. The role of bystander effects such as those described in Figure 1 and Figure 6c,d represent an interesting and often overlooked anti-tumor mechanism of oncolytic viruses. Cancer associated fibroblasts and other stromal cells significantly contribute to tumor biology, and may play a role in oncolytic virus therapy. Although the most potent death factor was produced by 3T6 Swiss albino fibroblast cell lines compared to the other fibroblast cancer cell lines tested, these results suggest a potential role for host fibroblasts in murine preclinical models of oncolytic virus therapy. HMGB1 release from fibroblast and other host and cancer cells might serve as a way to limit virus replication during lytic infection. HMGB1 release may also act as an adjuvant to standard chemotherapy drugs, as highlighted in Figure 6b and previous publications [7,35,44].

It is important to continue exploring novel ways to make oncolytic viruses more effective, and design strategies to use OVs and conventional chemotherapeutics in concert to increase the efficacy of current treatment modalities. Bolyard et al. showed doxorubicin and oncolytic HSV act synergistically in models of ovarian cancer [41]. Huang et al. have reported synergistic activity between oncolytic vaccinia virus and paclitaxel in a variety of tumor models in a HMGB1-dependent manner [17]. Our results herein demonstrate a significant difference in HSV1716 susceptibility between NIH-3T3 and 3T6 Swiss albino cell lines. We also show that HMGB1 secretion following virus infection can be toxic to cell lines in vitro, thereby limiting virus infection and spread. Finally, our data suggest that HMGB1 may play a role in the antiproliferative capacity of OVs alone or in combination with other therapies.

Despite derivation from NIH-3T3 cells, 3T6 cells bear significant differences from their 3T3 predecessors. For example, NIH-3T3 cells maintain contact inhibition while 3T6 cells continue to proliferate at high density [45]. The loss of contact inhibition in 3T6 cells, one of the hallmarks of malignant transformation, was later determined to be due in part to variances in the tumor suppressor p53 [46,47]. Many groups have reported on the interplay between p53 and the interferon response; indeed, the ability of HSV1716 and many other OVs to selectively infect malignant cells is predicated on aberrant antiviral responses in cancer cells [48,49,50,51]. Here, we show that NIH-3T3 cells resistant to HSV1716 infection actively secrete type I interferon following HSV1716 infection, while 3T6 Swiss albino cells susceptible to HSV1716 infection do not. This difference likely contributes to the differences in HSV1716gfp susceptibility we observed between NIH-3T3 and 3T6 Swiss albino cell lines. Over 50% of human tumors have p53 variations similar to those observed in 3T6 Swiss albino cells [52]. However, whether or not p53 status or interferon-mediated antiviral responses predict the susceptibility of cancers to OVs remains to be fully elucidated, and will likely be represented by a continuum of susceptibility.

Interferon-mediated JAK/STAT signaling activates hundreds of interferon stimulated genes (ISGs) during virus infection [53]. One of the many effects of JAK/STAT1 activation is HMGB1 translocation from the nucleus to the cytoplasm [25]. Workenhe et al. reported that oncolytic HSV stimulates robust HMGB1 secretion [31], consistent with our results. However, concomitant secretion of interferon is not required for HMGB1 release to occur, as indicated by our data in 3T6 Swiss albino cells infected with HSV1716. In the cytoplasm, HMGB1 binds a variety of molecules including foreign nucleic acids which are recognized by Toll-like receptors (TLRs) [26,54,55]. TLR signaling potentiates the antiviral response by driving inflammatory and immunostimulatory cytokine signaling, and initiating cell death [56,57,58]. Interestingly, HMGB1 been shown to augment TLR-9-mediated viral CpG DNA signaling [54]. The marked increase in virus spread and recovery in HMGB1 depleted 3T6 cells may be due in part to reducing the ability of HMGB1 to potentiate inflammatory responses that ultimately lead to cell death, as our western blot data (Figure 4c) and literature suggest [59]. It is plausible that by mitigating the direct and indirect cytotoxicity of virus, more viable cells are available to serve as viral replication factories. In line with this theory, previous reports show that blocking caspase activity in OV-infected cells leads to a more robust infection by limiting bystander cell death [60].

In addition to bolstering intracellular and extracellular signaling, HMGB1 also enhances cell cytotoxicity directly and indirectly [28,42,44]. HMGB1–DNA complexes, for example, have been implicated in the pathogenesis of systemic lupus erythematosus [61,62]. Other groups have reported cell death through a previously undescribed mechanism characterized by giant mitochondria formation when recombinant HMGB1 is added to glioblastoma and multiple other human cancer cell lines [42]. However, although we observe cytotoxicity with the addition of exogenous recombinant HMGB1 (Figure 7), it does not recapitulate the same antiproliferative effect as HMGB1-containing conditioned media from HSV1716gfp-infected 3T6 cells (Figure 6c,d). Exogenous recombinant HMGB1 was obtained from multiple sources to carry out gain of function studies in Figure 7, and it is important to note that HMGB1 from Sigma Aldrich yields two bands when probed via western blot, consistent with total cell lysates from cancer cell lines (Figure 5a), while R&D System’s HMGB1 protein migrates as one band on a polyacrylamide gel. Two bands for HMGB1 around 25/28 kDa is consistent with previous reports of reduced and oxidized HMGB1 isoforms, which may more accurately reflect physiologic forms of HMGB1 [43,55,63]. Further, although both NIH-3T3 and 3T6 Swiss albino cells secrete HMGB1 following HSV1716gfp infection, only conditioned media from 3T6 Swiss albino is cytotoxic. With its known propensity to bind other factors such as nucleic acids and cytokines, and the plethora of posttranslational modifications HMGB1 is subject to [55], we reason that HMGB1 toxicity is probably largely dependent on its binding partners and its posttranslational modifications. For example, Tang et al. reported that oxidized HMGB1 induced apoptosis in multiple cell lines, but the reduced form of HMGB1 did not [43]. In the context of an active virus infection, HMGB1 bound to viral nucleic acids could act as an adjuvant that ultimately exacerbates inflammatory responses thereby promoting cell death, immunity, and sensitization to antineoplastic drugs. Studying HMGB1 complexes in the context of OV therapy could unveil new ways of promoting inflammation and immunity within the tumor microenvironment.

## 5. Conclusions

HMGB1 can play many roles depending on its cellular context. Viral infection often induces HMGB1 secretion, as we show with NIH-3T3 and 3T6 Swiss albino cells infected with HSV1716. Here, we also showed that extracellular HMGB1 can impact virus spread, recovery, and act in an antiproliferative capacity during OV therapy alone or in combination with other forms of cancer treatment such as doxorubicin. Therefore, we conclude that HMGB1 plays a significant role in our previously observed VITA phenomenon [7,35]. However, this proof of concept requires further testing in a variety of tumor models. Exploring the diversity of HMGB1 complexes and posttranslational modifications could reveal new ways of promoting inflammation and immunity in OV therapy. Determining if HMGB1 modulation can enhance various cancer treatment modalities warrants further investigation and will likely vary on the cellular profile of the tumor microenvironment and tumor cell type itself.
